# The Effect of Oral Potentially Malignant Disorders (OPMD) on Dental Implants Survival—A Systematic Review

**DOI:** 10.3390/dj13010035

**Published:** 2025-01-15

**Authors:** Sebahat Kaya, Christian Walter, Aya Khamis, Lena Katharina Müller-Heupt, Stefanie Zimmer, Lorena Cascant Ortolano, Keyvan Sagheb, Bilal Al-Nawas, Eik Schiegnitz

**Affiliations:** 1 Department of Oral and Maxillofacial Surgery, University Medical Center, Johannes Gutenberg-University, 55131 Mainz, Germany; ayakhamis@uni-mainz.de (A.K.); keyvan.sagheb@unimedizin-mainz.de (K.S.); al-nawas@uni-mainz.de (B.A.-N.); eik.schiegnitz@unimedizin-mainz.de (E.S.); 2 Oral and Maxillofacial Surgery of the Mediplus Clinic, Haifa-Allee 20, 55128 Mainz, Germany; 3 Department of Otorhinolaryngology Head and Neck Surgery, Molecular and Cellular Oncology, University Medical Center, Johannes Gutenberg-University Mainz, 55131 Mainz, Germany; 4 Oral Pathology Department, Faculty of Dentistry, Alexandria University, Alexandria 5372066, Egypt; 5 Department of Periodontology and Operative Dentistry, University Medical Center, Johannes Gutenberg-University, 55131 Mainz, Germany; lena_katharina.mueller@unimedizin-mainz.de; 6 Institute of Pathology, University Medical Center, Johannes Gutenberg-University, 55131 Mainz, Germany; stefanie.zimmer2@unimedizin-mainz.de; 7 Departmental Library, University Medical Center Mainz, Johannes Gutenberg-University, 55131 Mainz, Germany; l.cascantortolano@ub.uni-mainz.de

**Keywords:** oral potentially malignant disorders, dental implants, complications, implant survival

## Abstract

**Objectives:** This research is purposed to synthesize the existing evidence on implant survival rates in patients with oral potentially malignant disorders (OPMD) and assess potential risk factors associated with peri-implant disease. **Material and Methods:** A comprehensive search was performed across PubMed MEDLINE, Cochrane Library, and Web of Science. This review was conducted according to the PRISMA guidelines, including studies published between 2012 and 2023. **Results:** The review of studies related to oral lichen planus (OLP) revealed an implant survival rate of 99.3% (50% to 100%) with a mean follow-up of 40.1 months. One retrospective study focused on patients with leukoplakia and erythroplakia, but did not provide data on implant survival; however, it reported the presence of oral squamous cell carcinomas (OSCC) in the vicinity of dental implants. Data from a patient with proliferative verrucous leukoplakia (PVL) indicated a 100% implant survival rate, while patients with systemic lupus erythematosus (SLE) showed an implant survival rate of 97.67%. For all other entities of OPMD no articles could be found. **Conclusions:** With the exception of OLP, there is a limited number of studies or none on all other entities of OPMD. The available literature indicates no impact of OLP on implant survival rates and does not support OLP as a risk factor for peri-implant disease. For the other entities of OPMD, no conclusion regarding implant survival or peri-implant disease risk factors can be drawn due to a lack of studies. To validate the results and evaluate OPMD on peri-implant tissue, large-scale prospective studies for all types of OPMD, especially for leukoplakia and erythroplakia, are needed.

## 1. Introduction

**Figure 1:**
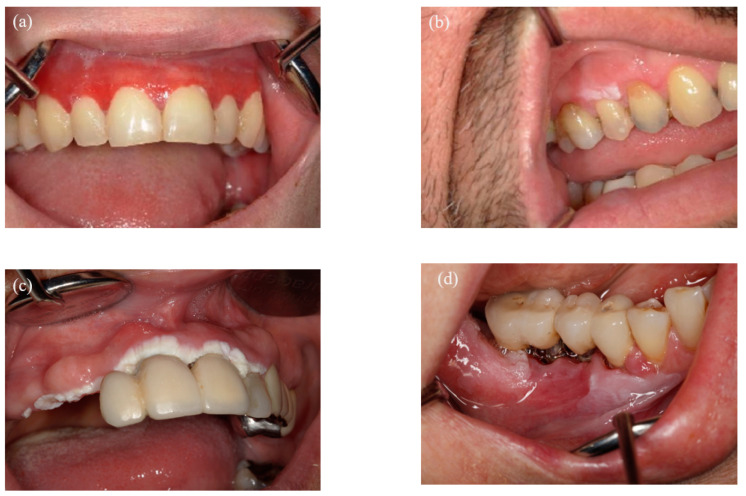
Clinical representation of OPMD entities: (**a**) oral lichen planus, (**b**) leukoplakia, (**c**) proliferative verrucous leukoplakia, (**d**) oral squamous cell carcinoma.

Oral potentially malignant disorders (OPMD) are a heterogeneous group of clinically defined mucosal disorders with epithelial lesions that are highly associated with the development of oral squamous cell carcinoma (OSCC) [1,2]. OPMD encompasses various entities, such as oral lichen planus (OLP), leukoplakia, proliferative verrucous leukoplakia (PVL), erythroplakia (EP), erythroleukoplakia (ELP), oral submucous fibrosis (OSF), actinic keratosis (AK), palatal lesions in reverse smokers, systemic lupus erythematosus (SLE), and dyskeratosis congenita (DKC) [2] (Figure 1). They form a heterogeneous group of oral lesions with varying clinical features, manifestations, risk factors, biological behavior, and rates of malignant transformation. OPMD are estimated to affect approximately 2% of the world population, with a 7.9% malignant transformation rate [3]. According to the WHO classification, epithelial dysplasia refers to a range of morphological and cytological changes in the epithelium resulting from the accumulation of genetic mutations [2], which increases the risk of malignant transformation [4]. Although epithelial dysplasia is postulated to be genetically triggered, the exact pathomechanism and their sequence are still quite unclear.

Mucosal diseases compromise the epithelial attachment to the implant surfaces [5,6]. Therefore, it has been suggested that when comparing healthy peri-implant mucosa to affected mucosa, there is a difference in response to bacterial infection as the diseased mucosa has a faster peri-implant soft tissue seal breakdown [7]. A transmucosal attachment is intended to prevent bacterial products from reaching the bone, thereby ensuring the successful osseointegration of the implant. To achieve this, a peri-implant soft tissue thickness of at least 2 mm is necessary [8].

Regarding OPMD, it has been assumed that the ability of adhesion to the titanium surface of implants would be jeopardized. Favorable results for the long-term success of dental implants depend primarily on the quality and quantity of the soft tissues and the bone. Factors influencing the soft tissues have various effects on bone loss and implant efficiency. The marginal bone around dental implants is often the primary site of bone loss [9], and the ability of the epithelial tissue to adhere and seal this area is a crucial factor for implant survival and function. For successful therapy with dental implants, the interaction between the upper part of the implant and the healthy oral mucosa is crucial to promote rapid epithelial cell adhesion and thus prevent inflammation after implantation [10].

It has been shown in initial studies that altered mechanical conditions due to mechanical stress and external mechanical influences, have in turn the potential to promote malignant degeneration [11]. The long-term impact of the interactions between dental implants and OPMD on the peri-implant soft tissue and bone remains unclear. Therefore, this systematic review aimed to assess the survival rate of dental implants in patients with OPMD and to identify potential risk factors for peri-implant diseases.

## 2. Material and Methods

### 2.1. Protocol Development and Eligibility Criteria

The study protocol was developed in accordance with the Preferred Reporting Items for Systematic Review and Meta-Analyses (PRISMA) guidelines. The research question was formulated using the Patient, Intervention, Comparison, and Outcome (PICO) framework, which can be specified as “Is there a difference between implant survival in patients with oral potential disorders and patients with non-OPMD”?

- Population: Patients with OPMD.
- Intervention: Implant surgery.
- Comparison: Patients with healthy mucosa.
- Outcome:
- Primary outcome: Implant survival.
- Secondary outcome: Risk factor for peri-implant disease.
- Peri-implant mucositis.
- Peri-implantitis.
- Bone loss.

The risk factors such as peri-implant mucositis, peri-implantitis, and bone loss were based on the definitions by the respective studies, as well as through clinical examination of the patients and radiological imaging.

### 2.2. Inclusion Criteria

1. Histopathologically or clinically confirmed OPMD (oral lichen planus, leukoplakia, proliferative verrucous leukoplakia, erythroplakia, systemic lupus erythematosus, and oral submucosal fibrosis).
2. Study published in English or German.
3. Mentioned number of implants.
4. Prospective studies: randomized controlled, non-randomized-controlled, and cohort studies.
5. Retrospective studies: controlled, case-control, single cohort, and case reports.

### 2.3. Exclusion Criteria

1. If a study did not fulfill all the criteria mentioned above or if essential information was missing and could not be supplied.
2. Studies without relevant data on implant survival or implant related outcomes.
3. Animal testing.
4. In vitro studies.

### 2.4. Search Strategy

The databases MEDLINE (via PubMed), the Cochrane Central Register of Controlled Trials (via Cochrane Library) and the Web of Science (Clarivate Analytics) were searched on 10 October 2023 for published literature on the research areas. A combination of Medical Subject Heading terms (MeSH) and free text terms for “dental implants” and “oral potentially malignant disorders” were identified. One of the search strategies performed in PubMed for MEDLINE is detailed below, with additional strategies available upon request by contacting the authors (Table 1).

To find additional potentially relevant articles, the reference lists of the relevant studies were also examined. Duplicates were eliminated using EndNote 20 (Clarivate Analytics, London, UK) by the librarian following the Bramer Method [12].

### 2.5. Study Selection

**Figure 2:**
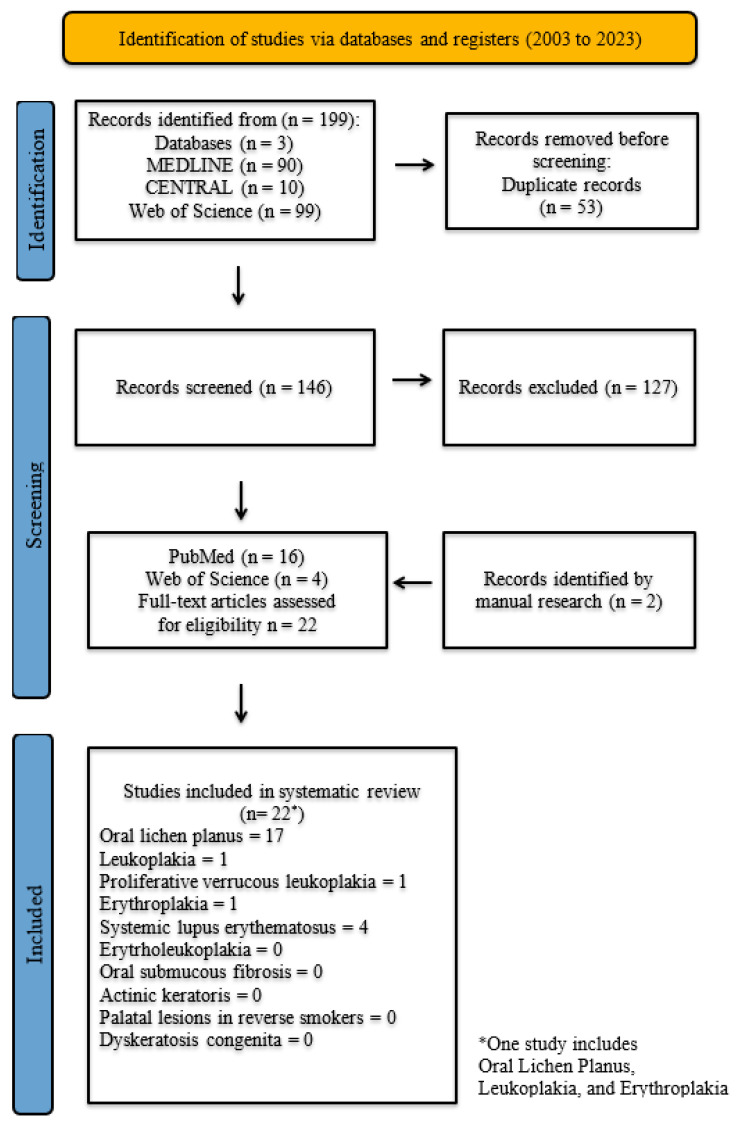
PRISMA flow diagram.

All studies that met the specified inclusion criteria were assessed in greater detail, and the full texts were acquired. The PRISMA flow diagram (Figure 2) illustrates the number of studies identified, excluded, and included.

### 2.6. Data Synthesis and Outcomes

The following information was extracted from each included study: the number (n) of patients, age, sex, number of implants, type of OPMD, biopsy of OPMD, peri-implant mucositis (PIM) and peri-implantitis (PI), bone loss, follow-up in months, implant survival, treatment of the OPMD before and/or after implant treatment, and malignant transformation. The primary outcome was implant survival. Secondary outcomes were the incidence of PIM, PI, and bone loss. The studies included in this review either made no statement regarding bone loss or the data were given quantitatively (in mm) or qualitatively (e.g., statements like crestal bone loss). In the case of quantitative data, no uniform measurements and statements are made. The following classification was used for the quantitative assessment of bone loss [13]:

- Bone loss < 3 mm.
- Bone loss ≥ 3 mm.

### 2.7. Assessment of Methodological Quality

The methodological quality of the included studies was evaluated using the Joanna Briggs Institute (JBI) critical appraisal tools for various study designs, including case reports, case series, case control studies, cross-sectional studies, and cohort studies [14]. The criteria for assessing methodological quality were based on the framework proposed by Goreth et al. [15]. For high methodological quality, a critical appraisal was required in which 80–100% of the questions were answered with “yes”. Studies classified as moderate quality answered 50–75% and studies classified as low quality answered 9–45% of the questions with “yes”.

## 3. Results

### 3.1. Study Selection

22 out of initially 199 articles were included in the review (Figure 2). Most articles were case reports [16,17,18,19,20,21,22,23,24,25,26,27,28,29], followed by six retrospective studies [30,31,32,33,34,35], one prospective study [36], and one cross-sectional study [37]. A total of 17 studies focused on OLP [16,17,18,19,20,21,22,26,27,29,30,31,32,33,34,36,37], four on systemic lupus erythematosus [24,25,28,35], and one study each was found for leukoplakia [31], proliferative verrucous leukoplakia [23], and erythroplakia [31]. One retrospective study of Moergel et al. [31] included patients with oral lichen planus, leukoplakia, and erythroplakia (Table 2). No eligible studies were found for erythroleukoplakia (ELP), oral submucous fibrosis, actinic keratosis (AK), palatal lesions in reverse smokers, and dyskeratosis congenita (DKC). A total of 3 studies compared implant survival rates in patients with OLP and healthy controls. Table S1 provides a summary of all included studies on implant survival rates in patients with OPMD, detailing their key findings and characteristics.

### 3.2. Oral Lichen Planus

A total of ten case reports [16,17,18,19,20,21,22,26,27,29], five retrospective studies [30,31,32,33,34], one prospective study [36], and one cross-sectional study [37] were included. A total of 365 implants in 153 patients have been described in these articles. The ages ranged from 44 to 83 years, with 84 female [54.90%], 27 male patients [17.65%], and 42 [27.45%] without a specified gender. For 300 out of the 365 implants a survival rate of 99.3% (50% to 100%) was reported, with two implants reported as lost (298/300) and a mean follow-up of 40.1 months.

Nine studies analyzed bone loss around the dental implants, either qualitatively [18,29,37] or quantitatively [17,22,27,33,34,36]. For 178 implants (48.76%), a quantitative assessment was provided, assessed radiographically in all cases [17,22,27,33,34], with one study using both radiographic and clinical measurements [36]. A bone loss of <3 mm was measured for 163 implants, while ≥3 mm was measured for 15 implants. In a total of 23/119 implants, a PI was measured [26,27,36,37], with one implant at 24 months [26], three implants at 12 months [27], five implants at 56.5 months [36], and fourteen implants at 42 months [37]. Similarly in 55/164 implants, a PIM was documented [17,32,36,37], with one implant at 36 months [17], nineteen implants at 36 months [32], twenty-five implants at 56.5 months [36], and ten implants at 42 months [37]. In the included studies, PIM and PI were reported based solely on their presence. One study used criteria by Roos-Jansåker et al. for PIM (BoP, PD ≥ 4 mm, no bone loss) and PI (BoP or pus, bone loss ≥ 3 threads) [36,38]. Another study assessed PIM as a binary variable (0 = no inflammation, 1 = signs such as redness, altered shape, or mucosal irregularities) with calibration to ensure consistency [37]

The perioperative (implant surgery) treatment of the OLP was described in nine articles: glucocorticoids with different active ingredients and different doses [20,22,29,30,32,33,34,36,37], retinoids [30], antibiotics, or chlorhexidine mouthwash [22]. In one study, 55 implants were inserted in 23 patients diagnosed with active OLP. Out of these, 42 implants failed, leading to a survival rate of 23.6%. Afterwards, patients were treated with low-energy soft tissue laser irradiation in ten sessions before implant placement. Furthermore, the dose of oral corticosteroids was gradually increased (5 mg every 10 days) until a daily dose of 20 mg was reached and maintained for 2 weeks. Subsequently, 42 implants were implanted, and after a 36 month follow-up, no implants were lost [32].

Three studies report an implant survival rate of 100% after a follow-up of 72 months [16], 36 months [17], and up to 24 months [30]. Anitua et al. reported an implant survival rate of 98.48% with a mean follow-up time of 68 months in 23 patients with 66 short implants. The loss of one implant in a patient with erosive OLP was observed [33]. One case report [22] and a case series [29] describe no implant loss in patients with OLP. In three studies, OLP patients were compared to healthy controls [34,36,37]. The first study, involving 18 patients with OLP, reported a survival rate of 100%, while the control group had a survival rate of 96.77% after a follow-up period of 56.5 months.

The prevalence of PIM in the control group was marginally higher (57%), whereas the prevalence of PI in the OLP group was greater (55.6%) [36]. Another study [37] found similar results, showing no significant differences between OLP patients and the control group in terms of implant survival, PI, PIM, and marginal bone loss. The overall success rate was 96.42% for the OLP group and 92% for the control group. The prevalence of PI and PIM prevalence in the OLP group was 17.86% and 25%, respectively, while in the control group, the prevalence was 18% and 16%, respectively. The last study analyzed the prognosis of implants in patients with OLP treated with low-dose systemic corticosteroids compared to non-controlled patients who continued oral corticosteroid therapy for another 12 weeks after implant placement and a healthy group. The study focused on marginal bone loss and observed no differences between healthy and controlled patients over a 4 year period. However, patients who were not controlled showed a significant increase in marginal bone loss [34].

Regarding malignant transformation, six studies [18,19,20,21,26,31] reported pathology of OSCC after placing the implants in nine patients (5.88%). It is important to mention that the main result of these six studies was the risk of malignancy associated with the presence of OLP and dental implants.

#### Assessment of the Methodological Quality of Studies for Oral Lichen Planus

For all studies of OLP, the methodological quality of the studies was assessed using the Joanna Briggs Institute (JBI) critical appraisal tool. All in all, critical appraisal tools were used for ten studies for case reports (Table 3), three studies for case series (Table 4), two studies for case control studies (Table 5), one study for a cross-sectional study (Table 6), and one study for a cohort study (Table 7). The data assessment showed four studies with a high quality (23%) [20,22,27,36], eleven studies with a moderate quality (65%) [17,18,19,21,26,29,30,31,32,34,37], and two studies with a low quality (12%) [16,33].

### 3.3. Leukoplakia

One study was included [31] that analyzed data on OLP, leukoplakia, and erythroplakia. It studied a total of twelve patients (six female and six male) aged between 42 and 88 years who developed OSCC around dental implants. The duration from implant placement to the onset of OSCC ranged from 29 to 120 months. No additional information was provided regarding the survival rate of the implants or the number of implants used. Moreover, there was no precise information on the time of implantation, the number of implants, or the exact follow-up time. However, it is important to note that the study examined cases of OSCC that developed in the vicinity of dental implants.

### 3.4. Proliferative Verrucous Leukoplakia

One case report was included [23] and described a 63 year-old female patient with a histologically diagnosed proliferative verrucous leukoplakia. A successful dental rehabilitation with implantation (follow-up 60 months) after multiple therapeutic procedures with cryosurgery, laser surgery, diathermic ablation, and excision surgery of the epithelial dysplasia and its malignant transformation was performed. Additional details regarding the number of implants, bone loss, and the survival rate of the implants were not given.

### 3.5. Erythroplakia

Although the database analysis identified five publications, none of these studies met the inclusion criteria. Nevertheless, in the study of Moergel et al. [31] two patient cases (one female 70 years, one male 73 years) with histopathologically diagnosed erythroplakia were rehabilitated with dental implants and 48 and 97 months after implant placement an OSCC was diagnosed, respectively.

### 3.6. Systemic Lupus Erythematosus

Three case reports [24,25,28] and one retrospective study [35] were included. A total of forty-three implants were placed in eight patients (five female, three male, and an age range from 28 to 66 years). The follow-up was between 18 and 58 months with an implant survival rate of 97.67% (42/43). In one study, the patient was treated with intravenous immunoglobulin every four weeks [28]. Another study investigated the use of dental implants with a calcium–ion-modified surface in combination with platelet concentrates for dental rehabilitation [35]. For 12 out of 43 implants (12/43; 27.90%) the bone loss was ≤3 mm and no further information was given [35].

### 3.7. Oral Submucous Fibrosis

No articles were included.

## 4. Discussion

The aim of this systematic review was to investigate the survival rates of dental implants in patients with OPMD and to evaluate possible risk factors associated with peri-implant diseases such as:

- Peri-implant mucositis.
- Peri-implantitis.
- Peri-implant bone loss.

Additionally, the potential for malignant transformation in patients with OPMD represents a significant risk factor. While malignant transformation was not explicitly defined as a secondary outcome in this review, its inclusion was deemed essential to comprehensively address the risks associated with OPMD. Given the complexity of managing implants in this patient population, we believe this broader approach enhances the clinical relevance and applicability of our findings.

Several prior reviews, including those by Torrejon-Moya et al. [39] and Chrcanovic et al. [40], have examined implant placement in the context of mucosal disorders; however, these studies predominantly focused on specific conditions, such as oral lichen planus, and on the general survival rates of implants. The recent systematic review by Li et al. [41] also investigates the relationship between oral lichen planus (OLP) and peri-implant diseases, though it does not address peri-implant bone loss.

In contrast, this systematic review represents the first comprehensive analysis of specific peri-implant risk factors commonly observed in patients with OPMD, including the development of PIM, PI, and peri-implant bone loss. Furthermore, our study underscores that the long-term progression of these conditions and the associated complications of their treatment remain inadequately explored in the existing literature, thus identifying an important avenue for future research.

### 4.1. Oral Lichen Planus

Oral lichen planus is defined as a chronic autoimmune inflammatory disease of unknown etiology with characteristic relapses and remissions showing white reticular lesions accompanied or not accompanied by atrophic, erosive, and ulcerative and/or plaque-like areas with frequently bilateral symmetric lesions [2].

In this systematic review, data from 365 implants placed in a total of 153 patients were analyzed. Generally, the implant survival rate in healthy individuals is reported to be around 95–98% after 5 to 10 years of follow-up [42,43], which aligns with the 99.3% (50–100%) implant survival rate in this review for patients with OLP. However, survival rates can vary depending on disease severity, treatment, and the patient’s response to implantation. Erosive OLP is painful and often resistant to treatment. Additionally, managing erosive OLP is highly challenging, and no gold standard treatment has been established thus far. However, several therapeutic approaches have shown effectiveness, including systemic corticosteroids, systemic retinoids, and anti-interleukin (IL)-17/anti-IL-23 drugs [44]. Although there is heterogeneity in the studies regarding symptomatic therapy in patients with OLP [20,22,29,30,32,33,34,36,37], the study by Aboushelib shows that implantation during the active phase of the disease can lead to complications and implant loss [32]. Furthermore, Anitua et al. reported that one implant failed due to episodes of inflammation in a patient with erosive disease and similarly concluded that peri-implant bone stability is reduced in the erosive form of OLP [33]. These findings suggest that implant placement should be avoided during the acute phase of OLP, as complications like implant loss are more likely, and implants can be successfully placed once the disease is under control. It is therefore recommended to avoid the insertion of implants during the acute phase of the disease due to the higher risk of inflammation [41].

Peri-implant bone loss is an indicator of the risk for long-term complications around the implant, including a significantly increased risk of PI, which can eventually lead to the loss of osseointegration [45]. While the literature describes the risk for PI at early bone loss thresholds as low as 0.5 mm and 1 mm [46], in daily clinical practice, PI is defined as a bone loss ≥3 mm [13]. However, there is a lack of consistency in the reported thresholds across studies. To address this variability, we chose to classify bone loss into two categories: <3 mm and ≥3 mm. This classification provides a structured approach to comparing the diverse data while distinguishing between early and advanced bone loss.

In our analysis, a bone loss of ≥3 mm was reported in four studies, involving 15 implants [17,22,27,36]. In addition, PI was observed in 23 out of 119 implants [26,27,36,37], while PIM was documented in 55 out of 164 implants [17,32,36,37]. The study by Hernandez et al. provides further insights into these outcomes, suggesting that the higher prevalence of PIM in the control group (58%) compared to the OLP group (44.6%) may be attributed to the excellent oral hygiene maintained by patients with OLP [36]. The studies suggest that risk factors such as bone loss, PIM, and PI do not significantly impact the long-term success of dental implants in patients with OLP. PIM and PI rates in OLP patients (17.86% and 25%, respectively) are comparable to those in the general population (18% and 16%) [37].

However, the studies revealed significant heterogeneity in the localization of OLP within the oral cavity. It remains unclear whether OLP has a direct impact on peri-implant tissue. Only two studies mentioned the occurrence of OLP near implants [17,26]. Another study at least mentioned that complete healing of the localization of the implant was a prerequisite for the study [36]. In some studies, there were isolated clinical images suggestive of active OLP manifestations around peri-implant tissue [21,29,30,32,37].

Malignant transformation was diagnosed in a total of nine patients with dental implants [18,19,20,21,26,31]. Moergel et al. analyzed the risk of oral squamous cell carcinoma in patients with OLP and dental implants, suggesting that OLP patients may be at elevated risk of OSCC, particularly in areas exposed to chronic irritation, such as near dental implants [31]. However, a direct link between dental implants and malignant transformation remains unclear. Similarly, case reports by Noguchi et al. and Martin-Cabezas et al. highlight potential risks, including epithelial hyperplasia and changes around implants that may mimic PIM [26,27]. It is hypothesized that implant placement may contribute to the development of OSCC originating from the periodontal epithelium as a result of periodontal tissue and damage and the loss of the periodontal ligament [47].

Within the limitations of the current evaluation and aware of possible biases of the included studies, the present review supports the existing recommendation to provide patients with OLP the option of implant therapy. Nevertheless, no patient should be treated with dental implants during an acute phase of exacerbation, especially in symptomatic and/or erosive OLP. Careful oral hygiene and regular guideline-compliant screening of the oral mucosa are important to prevent an inflammatory tissue reaction and malignant transformation, or to diagnose early.

### 4.2. Leukoplakia

According to the WHO classification, leukoplakia is considered a “predominantly white plaque of questionable risk having excluded (other) known diseases or disorders that carry no increased risk for cancer defines” [48] with a worldwide prevalence of about 4.1% [49]. Nonhomogeneous leukoplakia shows a higher risk of malignant transformation compared to the homogeneous form which can be pronounced with epithelial dysplasia of varying severity [9]. Moergel et al. [31], who reported the largest series of cases of cancer near dental implants, highlighted PIM as the predominant clinical sign in 12 cases of oral leukoplakia. While the incidence of malignant tumors near dental implants remains very low, it is still unclear whether interactions between implant and peri-implant tissues play an important role in carcinogenesis. Nevertheless, mechanical irritation is thought to be at least a cofactor in oral carcinogenesis [50]. A recent meta-analysis demonstrated this association between chronic oral mucosal irritation and OSCC, and chronic mechanical irritation may act as a potential cofactor [51]. Our literature research has shown that there are no studies and data available that have investigated the prognosis, complications, and risk of malignant transformation in patients with leukoplakia and rehabilitation with dental implants.

### 4.3. Proliferative Verrucous Leukoplakia

Proliferative verrucous leukoplakia is defined as a progressive, persistent and irreversible form of oral leukoplakia characterized by a high risk of malignant transformation, with a cumulative malignant transformation rate of 49.5% [3]. There are no relevant data and studies on the survival rate of dental implants and complications leading to malignant transformation in their vicinity. Due to the high risk of malignant transformation, early diagnosis, surgical removal, and long-term guideline-compliant follow-ups are the keys to success for patients with proliferative verrucous leukoplakia.

### 4.4. Erythroplakia

Erythroplakia is an inhomogeneous leukoplakia defined as a predominantly fiery red patch that cannot be characterized clinically or pathologically as any other definable disease [2]. Erythroplakia has the second highest malignant transformation rate with 33.1% of all OPMD right after the proliferative verrucous leukoplakia [3]. Only one retrospective study with two patients was included in this systematic review. Moergel et al. reported two cases of OSCC associated with erythroplakia and leukoplakia around dental implants, both involving patients with a history of oral cancer [31]. Due to the insufficient data and studies on patients with erythroplakia who have been rehabilitated with dental implants, no evidence-based recommendation can be made regarding the implant success rate and complications. In summary, the focus should also be on the early detection of erythroplakia as well as surgical excision and long-term guideline-compliant aftercare.

### 4.5. Systemic Lupus Erythematosus

Systemic lupus erythematosus is a chronic autoimmune disease, which can be principally subdivided into systemic, drug-induced, and discoid forms [2]. Approximately 20% of patients with systemic lupus erythematosus develop oral manifestations in the course of their disease, which exhibit similar clinical presentations as found in oral lichen planus [2]. Only three case report studies [24,25,28] and one retrospective study [35] with data on eight patients with systemic lupus erythematosus were found. In patients with systemic lupus erythematosus, wearing mucosal prostheses is impaired due to oral ulcers and hyposalivation [52,53]. In addition, ill-fitting prostheses or traumatic interactions with the mucosal tissue can lead to ulceration [54]. Patients with systemic lupus erythematosus would therefore benefit from implant-supported or implant-fixed prosthetic treatment. In patients with systemic autoimmune diseases with manifestations of the oral mucosa, as well as patients with OPMD, a careful follow-up should always be performed whenever possible.

## 5. Conclusions

- The implant survival rate in patients with oral lichen planus (OLP) is 99.3%, comparable to healthy individuals.
- Bone loss of 3 mm or more is a critical risk factor for peri-implantitis and should be regularly monitored to prevent long-term complications.
- Implant placement should be avoided during the acute phase and should only be placed once the OLP condition is stable to minimize risks.
- Peri-implant tissues play a pivotal role in implant success, emphasizing the need for preventive measures both before and after treatment.
- The risk of malignant transformation in OPMD patients remains a significant concern, requiring further investigation to optimize monitoring protocols.
- The diverse etiologies and clinical manifestations of OPMD present challenges for implant therapy, highlighting the importance of individualized treatment strategies and adherence to guidelines.
- Clinical data on OPMDs beyond OLP are limited, making it difficult to draw definitive conclusions. More prospective studies focusing on lesion types, locations, and patient-specific factors are needed.
- Standardized diagnostic criteria for peri-implant diseases are necessary to improve consistency in future studies.
- Future research should include controlled, multicenter studies with extended follow-up periods to validate findings and improve their applicability.

## Figures and Tables

**Table 1:** Search strategy on 10 October 2023.

#1	(“dental implant*”[tiab] OR “dental prosthes*”[tiab] OR “oral implant*”[tiab] OR “dental implants”[Mesh] OR “dental implantation”[Mesh] OR “dental prosthesis, implant supported”[Mesh]).
#2	(“Precancerous Conditions”[Mesh:NoExp] OR “oral potentially malignant disorder*”[tiab] OR OPMDS[tiab] OR OPMD[tiab] OR “preneoplastic condition*”[tiab] OR “precancerous condition*”[tiab] OR (leukoplakia[Mesh] OR leukoplaki*[tiab] OR leucoplaki*[tiab] OR “oral dysplasi*”[tiab] OR “oral keratos*”[tiab]) OR (erythroplasia[Mesh] OR erythroplasi*[tiab] OR erythroplaki*[tiab]) OR (“lichen planus, oral”[Mesh] OR “oral lichen planus”[tiab] OR OLP[tiab]) OR (“oral submucous fibrosis”[Mesh] OR “oral submucous fibros*”[tiab] OR OSF[tiab]) OR (“libman sacks diseas*”[tiab] OR “lupus erythematosus disseminatus”[tiab] OR “systemic lupus erythematosus”[tiab] OR SLE[tiab] OR “lupus erythematosus, systemic”[Mesh:NoExp]) OR (“actinic keratos*”[tiab] OR “keratosis, actinic”[Mesh] OR AK[tiab]) OR “reverse smok*”[tiab] OR (“dyskeratosis congenita*”[tiab] OR “zinsser cole engman syndrom*”[tiab] OR “Dyskeratosis Congenita”[Mesh])).
#3	#1 and #2.

**Table 2:** Summary of studies, number of patients and implants, PIM, PI, bone loss, follow-up, implant survival, implant success, and malignant transformation in all entities of OPMD.

OPMD	Oral Lichen Planus	Leukoplakia	Proliferative Verrucous Leukoplakia	Erytrhoplakia	Systemic Lupus Erythematosus
Studies	17	1	1	1	4
Patients	153	12	1	2	8
Implants	365	Nm	Nm	Nm	43
PIM	55/164	Nm	Nm	Nm	Nm
PI	23/119	1/12	Nm	Nm	Nm
Bone loss <3 mm	163/178	Nm	Nm	Nm	12/43
Bone loss ≥3 mm	15/178	Nm	Nm	Nm	x
Mean follow-up (months)	40.1	65.25	60	72.5	34
Implant survival	99.33% (298/300)	Nm	100%	Nm	97.67%
Implant success	96.42% (56/56)	Nm	Nm	Nm	Nm
Malignant Transformation	9	12	Nm	2	0

PIM: peri-implant mucositis; PI: peri-implantitis; Nm: not metioned.

**Table 3:** Critical appraisal results of case reports.

Assessment of Studies Using the Joanna Briggs Institute Critical Appraisal Tools for Case Reports									
Study	Were Patient’s Demographic Characteristics Clearly Described?	Was the Patient’s History Clearly Described and Presented as a Timeline?	Was the Current Clinical Condition of the Patient on Presentation Clearly Described?	Were Diagnostic Tests or Assessment Methods and the Results Clearly Described?	Was the Intervention(s) or Treatment Procedure(s) Clearly Described?	Was the Post-Intervention Clinical Condition Clearly Described?	Were Adverse Events (Harms) or Unanticipated Events Identified and Described?	Does the Case Report Provide Takeaway Lessons?	Assessment of Methodological Quality
Esposito et al. (2003) [29]	Yes	Yes	No	Yes	No	No	No	Yes	Moderate quality
Öczakir et al. (2005) [16]	Yes	No	No	No	No	No	No	Yes	Low quality
Reichart (2006) [17]	Yes	Yes	Yes	No	Yes	No	No	Yes	Moderate quality
Czerninski et al. (2006) [18]	Yes	Yes	No	Yes	No	No	No	Yes	Moderate quality
Gallego et al. (2008) [19]	Yes	Yes	No	Yes	No	No	No	Yes	Moderate quality
Marini et al. (2013) [20]	Yes	Yes	Yes	Yes	Yes	No	Yes	Yes	High quality
Raiser et al. (2016) [21]	Yes	No	Yes	Yes	Yes	No	Yes	Yes	Moderate quality
Fu et al. (2019) [22]	Yes	Yes	Yes	Yes	Yes	Yes	Yes	Yes	High quality
Noguchi et al. (2019) [26]	Yes	Yes	Yes	Yes	No	No	No	Yes	Moderate quality
Martin-Cabezas et al. (2021) [27]	Yes	Yes	Yes	Yes	Yes	Yes	Yes	Yes	High quality

**Table 4:** Critical appraisal results of case series.

Assessment of Studies Using the Joanna Briggs Institute Critical Appraisal Tools for Case Series											
Study	Were There Clear Criteria for Inclusion in the Case Series?	Was the Condition Measured in a Standard, Reliable Way for All Par-Ticipants Included in the Case Series?	Were Valid Methods Used for Identificaton of the Condition for All Participants Included in the Case Series?	Did the Case Series Have Consecutive Inclusion of Participants?	Did the Case Series Have Complete Inclusion of Participants?	Was There Clear Reporting of the Demographic of the Participants in the Study?	Was There Clear Reporting of Clinical Information of the Participants?	Were the Outcomes or Follow up Results of Cases Clearly Reported?	Was There Clear Reporting of the Presenting Site(s)/ Clinic(s) Demographic Information?	Was Statistical Analysis Appro- Priate?	Assessment of Metho- Dological Quality
Moergel et al. (2014) [31]	No	Yes	Yes	Yes	Unclear	Yes	Yes	Not applicable	Not applicable	Yes	Moderate quality
Aboushelib et al. (2017) [32]	No	Yes	Yes	No	Unclear	Yes	No	Yes	Not applicable	Yes	Moderate quality
Anitua et al (2018) [33]	Yes	No	Yes	No	Unclear	No	No	Yes	Not applicable	Yes	Low quality

**Table 5:** Critical appraisal results of case control studies.

Assessment of Studies Using the Joanna Briggs Institute Critical Appraisal Tools for Case Control Studies											
Study	Were the Groups Comparable Other Than the Presence of Disease in Cases or the Absence of Disease in Controls?	Were Cases and Controls Matched Appropriately?	Were the Same Criteria Used for Identification of Cases and Controls?	Was Exposure Measured in a Standard, Valid and Reliable way?	Was Exposure Measured in the Same Way for Cases and Controls?	Were Confounding Factors Identified?	Were Strategies to Deal with Confounding Factors Stated?	Were Outcomes Assessed in Standard, Valid and Reliable Way for Cases and Controls?	Was the Exposure Period of Interest Long Enough to be Meaningful?	Was Appropiate Statistical Analysis Used?	Assessment of Metho- Dological Quality
Hernandez et al. (2012) [36]	Yes	Yes	Yes	Yes	Yes	No	Not applicable	Yes	Yes	Yes	High quality
Czerninski et al. (2013) [30]	Yes	Yes	Yes	Yes	Yes	No	Not applicable	Yes	No	Yes	Moderate quality

**Table 6:** Critical appraisal results of cross-sectional study.

Assessment of Studies Using the Joanna Briggs Institute Critical Appraisal Tools for Analytical Cross Sectional Study									
Study	Were the Criteria for Inclusion in the Sample Clearly Defined?	Were the Study Subjects and the Setting Described in Detail?	Was the Exposure Measured in a Valid an Reliable Way?	Were Objective, Standard Criteria Used for Measurement of the Condition?	Were Confounding Factors Identified?	Were Strategies to Deal with Confounding Factors Stated?	Were the Outcomes Measured in a Valid and Reliable Way?	Was Appropriate Statistical Analysis Used?	Assessment of Methodological Quality
Lopez-Jornet et al. (2014) [37]	Yes	Yes	Yes	No	Unclear	Unclear	Yes	Yes	Moderate quality

**Table 7:** Critical appraisal results of cohort study.

Assessment of Studies Using the Joanna Briggs Institute Critical Appraisal Tools for Cohort Studies												
Study	Were the Two Groups Similar and Recruitedfrom the Same Opulation?	Were the Exposure Measured Similarly to Assign People to Both Exposed and Unexposed Groups?	Was the Exposure Measured in a Valid and Reliable Way?	Were Confounding Factors Identified?	Were Strategies to Deal with Confoun-ding Factors Stated?	Were the Groups/Participants Free of the Outcome at the Start of the Study (or at the Moment of Exposure)?	Were the Outcomes Measured in a Valid and Reliable Way?	Was the Follow up Time Reported and Sufficient to Be Long Enough or Outcomes to Occur?	Was Follow up Complete, and if Not, Were the Reasons to Loss to Follow up Described and Explored?	Were Strategies to Address Incomplete Follow up Utilized?	Was Appropriate Statistical Analysis Used?	Assessment of Metho-dological Quality
Khamis et al. (2019) [34]	No	Yes	No	No	No	No	Yes	Yes	Yes	No	Yes	Moderate quality

## Supplementary Materials

The following supporting information can be downloaded at: https://www.mdpi.com/article/10.3390/dj13010035/s1, Table S1. Summary of studies on implant survival rate in patients with OPMD [16,17,18,19,20,21,22,23,24,25,26,27,28,29,30,31,32,33,34,35,36,37].

## Abbreviations

OLP	Oral Lichen planus
OPMD	Oral Potentially Malignant Disorders
OSCC	Oral Squamous Cell Carcinoma
PI	Peri-implantitis
PIM	Peri-implant mucositis
WHO	World Health Organization
Fig.	Figure
Na	Not applicable
Nm	Not mentioned
