# Supplementary material for: The Effect of Oral Potentially Malignant Disorders (OPMD) on Dental Implants Survival—A Systematic Review
Source: Dent J (Basel). 2025 Jan 15;13(1):35. doi: 10.3390/dj13010035 (PMC11763872; doi:10.3390/dj13010035)
Supplement: Supplementary file 1 [file dentistry-13-00035-s001.zip › dentistry-3268020-supplementary.pdf]

dentistry journal

MDPI

**Table S1.** Summary of studies on implant survival rate in patients with OPMD.

| Study | Study type | No. of patients age/mean age | Gender | No. of implants | Type | OPMD Biopsy | PIM PI | OPMD in the peri-implant environment | Bone loss (mm) for each implant | Implant survival / Follow-up (months) | Treatment | OSCC | Outcome |
|---|---|---|---|---|---|---|---|---|---|---|---|---|---|
| **Oral Lichen Planus** | | | | | | | | | | | | | |
| Esposito et al. (2003) [29] | Case Report | 2<br>72<br>78 | F | 4 | Erosive | Yes | Nm | Nc | 4× minimal crestal | 100% / 21 | Topical steroids after implant placement for two weeks | Nm | Implant success |
| Öczakir et al. (2005) [16] | Case Report in a case series study | 1<br>74 | F | 4 | Nm | Nm | No | No | 4× Not measured | 100% / 72 | No | Nm | Implant survival |
| Reichart (2006) [17] | Case Report | 3<br>63<br>68<br>79 | F | 4<br>1<br>5 | Reticular<br>Reticular<br>Atrophic | Nm | 1× PIM | Yes | 2× 3–4 mm, 2× Nm 1× first thread of the implant 5× 1–3 mm | 100% / 36 | No | Nm | Implant survival |
| Czerninski et al. (2006) [18] | Case Report | 1<br>52 | F | 3 | Severe manifestations | Nm | No | No | 2× crestal | Nm / 36 | No | Yes | Risk for malignancy |
| Gallego et al. (2008) [19] | Case Report | 1<br>81 | F | 2 | Erosive Plaque | Yes | No | No | 2× Nm | Nm / 36 | No | Yes | Risk for malignancy |
| | | 18 | | 56 | Erosive | Yes | | Nc | | 100% | | Nm | |

| | | | | | | | | | | | | | |
|---|---|---|---|---|---|---|---|---|---|---|---|---|---|
| Hernandez et al. (2012) [36] | Prospective-controlled | mean age 53.7 | 14F 4M | | | | PIM 25/56 PI 5/56 | | ≤ 1.7→18/32.1 % 1.8–2.4→24/42.9 % 2.5–3→8/14.3% 3.1–3.6→4/7.1% ≥3.7→2/3.6% | 56.5 median | Clobetasol propionate 0.05%, 3t/d (18 patients) + prednisone 30 mg 1t/d 5–10d (2 patients) | | Implant failure |
| | | | | | | | | | | 96.77% | | | |
| | | Control-group: 18 Mean age 52.2 | 12F 6M | 62 | | | PIM 36/62 PI 4/62 | Nc | ≤ 1.7→22/36.7 % 1.8–2.4→24/4% 2.5–3→9/15% 3.1–3.6→3/5% ≥3.7→2/3.3% | 52.5 median | | | |
| Czerninski et al. (2013) [30] | Retrospective | 14 OLP patients with dental implant mean age 59.5 | 11F 3M | 54 | Reticular Erythematous Atrophic Erosive | Yes | No | Nc | 54× Nm | 100% | Retinoid 0.05/0.025% Triamcinolone 0.1% Clobetasol propionate 0.05% | Nm | Implant survival |

| | | | | | | | | | | | | | |
|---|---|---|---|---|---|---|---|---|---|---|---|---|---|
| | | 15 OLP patients without dental implant | | | | | | | | 12–24 | Dexamethasone 0.4%/Triamcinolon e 8mg | | |
| Marini et al. (2013) [20] | Case Report | 1<br>51 | F | 2 | Plaque | Nm | No | No | 2× Nm | 50%<br>48 | Prednisone, 30 mg daily (before Implantation) | Yes | Risk for malignancy in the presence of OLP and dental implant |
| Moergel et al. (2014) [31] | Retrospectiv e | 3<br>54<br>69<br>80 | F | Nm | Nm | Yes | No | No | Nm | Nm<br>6–51 mean 21.33 (time to cancer) | Nm | Yes | Potential risk factors for carcinomas in the vicinity of dental implants |
| Lopez-Jornet et al. (2014) [37] | Cross-sectional | 16 mean age 64.5 (44–76) | 10F 6M | 56 | 11 Reticular-Papular<br>5 Atrophic-Erosive | Yes | PIM 10/56<br>PI 14/56 | Nc | 10/56 | 96.42% (success)<br>42 (12–120) | Topical corticosteroids (0.01% Triamcinolone acetoniode) 3t/day | Nm | Implant survival |
| | | 16 mean age 42 (29–79) | 8F 8M | 50 | Nm | | PIM 9/50<br>PI 8/50 | Na | 8/50 | 92%<br>48 (24–48) | | | |
| Raiser et al. (2016) [21] | Case Report | 2<br>55<br>70 | F | Nm | Nm | Nm | No | Nc | 1xPatient: substantial<br>1x Patient: horizontal | Nm<br>Nm | Nm | Yes | Primary malignancy of the peri-implant mucosa |

| | | | | | | | | | | | | | |
|---|---|---|---|---|---|---|---|---|---|---|---|---|---|
| Aboushelib et al. (2017) [32] | Retrospective | 23 mean age 56.7 | 12F 11M | First set of implants: 55<br>Second set of implants: 42 | Nm | Yes | Nm<br>PIM on 19 implants | Nc<br>Nc | 55× Nm<br>42× initial resorption of crestal bone (0.26 mm ± 0.32) | First set of implants: 23.6%<br>First set of implants: 3<br>Second set of implants: 100%<br>Second set of implants: 36 | First set of implants: No<br>Second set of implants: Oral corticosteroid: 5 mg/day for 10 days 10 mg /day for 10 days 15 mg/day for 10 days 20 mg/day for 21 days and further for 14 days; after than reduce the dose by 5 mg every 10 days Diode Laser: 10 sessions of low-energy soft tissue laser irradiation | Nm | Implant survival Implant success |
| Anitua et al. (2018) [33] | Retrospective | 23 mean age 58 | 20F 3M | 66 (Reticular: 40; Erosive: 26) | 15 Reticular 8 Erosive | Yes | Nm | No | 66× mean bone loss 0.96 mm mesially, 0.99 mm distally | Reticular: 100% Erosive: 96.2% 25/26 →98.48%<br>24–124 68 mean | Deflazacort 30 mg 2 days preoperative, 15 mg 3 days postoperatively, 7.5 mg for another 3 days | Nm | Implant survival Marginal bone loss |

| | | | | | | | | | | | | | |
|---|---|---|---|---|---|---|---|---|---|---|---|---|---|
| Fu et al. (2019) [22] | Case Report | 1<br>65 | F | 4 | Erosive | Yes | PIM: No<br>PI: Nm | No | 4× 3–4 mm | 100%<br>36 | Before implantation: oral corticosteroids After implantation: Amoxicillin 1 g 1 h before implant placement and continuing for 7 days (1 g/12 h), chlorhexidine mouthwash twice daily for 14 days, topical corticosteroids (0.01% triamcinolone acetonide 3t/days until remission) | Nm | Implant survival |
| Khamis et al. (2019) [34] | Retrospective cohort | controlled lichen planus patients: 20 | Nm | 20 | Reticular, Plaque,Papular, Atrophic/Erosive, Bullous, Ulcerative | Yes | Nm | No | 20× controlled: 0.75 mm | 100%<br>48 | Diode soft tissue laser sessions, systemic corticosteroids 4 weeks before implant placement and continued 4 mg/48 hrs | Nm | Marginal bone loss Implant survival |
| | | | | 22 | | | | | | 100% | | | |

| | | | | | | | | | | | | | |
|---|---|---|---|---|---|---|---|---|---|---|---|---|---|
| | | non-controlled lichen planus patients: 22 | | | | | | | 22× non-controlled: 2.53 mm | 48 | Diode soft tissue laser sessions, systemic corticosteroids 4 weeks before implant placement and 12 weeks after implant placement | | |
| | | healthy: 17 | | 17 | | | | | 17× healthy: 0.79 mm | 100%<br>48 | | | |
| Noguchi et al. (2019) [26] | Case report | 1<br>78 | F | 4 | Nm | Yes | PI on 1 Implant | Yes | Nm | Nm<br>24 | Nm | Yes | Risk for malignancy |
| Martin-Cabezas et al. (2021) [27] | Case report | 1<br>83 | F | 3 | Erosive | Nm | PI on 3 Implants | No | ≥3 mm on each implant | 100%<br>12 | Initial and surgical peri-implant therapy | Nm | Influence of peri-implant healing Implant survival |
| **Leukoplakie** | | | | | | | | | | | | | |
| Moergel et al. (2014) [31] | Retrospective | 12 mean age 68.41 | 6F 6M | Nm | Nm | Yes | PI 1× | No | Nm | Nm<br>29–120 mean 65.25 (time to cancer) | Nm | Yes | Potential risk factors for carcinomas in the vicinity of dental implants |
| **Proliferative Verrucous Leukoplakia** | | | | | | | | | | | | | |
| | Case Report | 1 | F | Nm | Na | Yes | Nm | No | Nm | 100% | | No | Quality of life |

| | | | | | | | | | | | | | |
|---|---|---|---|---|---|---|---|---|---|---|---|---|---|
| Capodiferro et al. (2005) [23] | | 63 | | | | | | | | 60 | Cryosurgery, laser surgery, diathermic ablation, excisional surgery | | |
| **Erythroplakia** | | | | | | | | | | | | | |
| Moergel et al. (2014) [31] | Retrospective | 2<br>70<br>73 | 1F<br>1M | Nm | Nm | Yes | Nm | No | Nm | Nm<br>48–97 mean 72.5 (time to cancer) | Nm | Yes | Potential risk factors for carcinomas in the vicinity of dental implants |
| **Oral Lupus Erythematosus** | | | | | | | | | | | | | |
| Ergun et al. (2010) [24] | Case Report | 1<br>49 | F | 6 | Na | Nm | Nm | Nm | Nm | 100%<br>24 | Nm | No | Implant supported oral rehabilitation |
| Todorovic et al. (2017) [25] | Case Report | 1<br>66 | F | 10 | Na | Nm | No | Nm | No | 100%<br>36 | Nm | No | Oral rehabilitation with implant-supported fixed dentures |
| Drew et al. (2018) [28] | Case report | 1<br>28 | F | 15 | Na | Nm | Nm | Nm | Nm | 93.3%<br>18 | Intravenous immunoglobulin every 4 weeks | Nm | Survival rate of implants and suprastructure |
| Mozzati et al. | Retrospective | 5 | 2F<br>3M | 12 | Na | Nm | PIM: No<br>PI: Nm | No | 12 × 0.49 mm ± 0.18 mm | 100% | Preoperative: Professional oral | No | Consisting of a calcium |

| | | | | | | | | | | |
|---|---|---|---|---|---|---|---|---|---|---|
| (2021) [35] | 50.70 mean | | | | | | | 58.47 mean | hygiene, Amoxicillin 1g every 12 hours for 5 days Bioactivation of implants with plasma Postoperatively: oral hygiene with 0.2% chlorhexidine solution for 14 days | ions deposition over the implants |

## References


1. Oczakir, C.; Balmer, S.; Mericske-Stern, R. Implant-prosthodontic treatment for special care patients: A case series study. *Int. J. Prosthodont.* **2005**, *18*, 383–389.
2. Reichart, P.A. Oral lichen planus and dental implants. Report of 3 cases. *Int. J. Oral Maxillofac. Surg.* **2006**, *35*, 237–240.
3. Czerninski, R.; Kaplan, I.; Almoznino, G.; Maly, A.; Regev, E. Oral squamous cell carcinoma around dental implants. *Quintessence Int.* **2006**, *37*, 707–711.
4. Gallego, L.; Junquera, L.; Baladrón, J.; Villarreal, P. Oral squamous cell carcinoma associated with symphyseal dental implants: An unusual case report. *J. Am. Dent. Assoc.* **2008**, *139*, 1061–1065.
5. Marini, E.; Spink, M.J.; Messina, A.M. Peri-implant primary squamous cell carcinoma: A case report with 5 years' follow-up. *J. Oral Maxillofac. Surg.* **2013**, *71*, 322–326.
6. Raiser, V.; Abu-El Naaj, I.; Shlomi, B.; Fliss, D.M.; Kaplan, I. Primary Oral Malignancy Imitating Peri-Implantitis. *J. Oral Maxillofac. Surg.* **2016**, *74*, 1383–1390.
7. Fu, L.; Liu, Y.; Zhou, J.; Zhou, Y. Implant-Retained Overdenture for a Patient With Severe Lichen Planus: A Case Report With 3 Years' Follow-Up and a Systematic Review. *J. Oral Maxillofac. Surg.* **2019**, *77*, 59–69.
8. Capodiferro, S.; Maiorano, E.; Di Cosola, M.; Lacaita, M.G.; Lo Muzio, L.; Favia, G. Proliferative verrucous leukoplakia: May dental rehabilitation with implants allow better quality of life and prolong disease-free intervals? *Oral Oncol.* **2005**, *1*, 204.
9. Ergun, S.; Katz, J.; Cifter, E.D.; Koray, M.; Esen, B.A.; Tanyeri, H. Implant-supported oral rehabilitation of a patient with systemic lupus erythematosus: Case report and review of the literature. *Quintessence Int.* **2010**, *41*, 863–867.
10. Todorovic, V.S.; Milic, M.S.; Vasovic, M.; Nikolic, Z. Oral rehabilitation of a patient with systemic lupus erythematosus using implant-supported fixed dentures—A case report with review of important considerations. *Srp. Ark. Celok. Lek.* **2018**, *146*, 567–571.
11. Noguchi, K.; Moridera, K.; Sotsuka, Y.; Yamanegi, K.; Takaoka, K.; Kishimoto, H. Oral squamous cell carcinoma occurring secondary to oral lichen planus around the dental implant: A case report. *Oral Sci. Int.* **2019**, *16*, 110–113.

12. Martin-Cabezas, R. Peri-implantitis management in a patient with erosive oral lichen planus. A case report. *Clin. Case Rep.* **2021**, *9*, 718–724.
13. Drew, A.; Bittner, N.; Florin, W.; Koch, A. Prosthetically Driven Therapy for a Patient With Systemic Lupus Erythematosus and Common Variable Immunodeficiency: A Case Report. *J. Oral Implantol.* **2018**, *44*, 447–455.
14. Esposito, S.J.; Camisa, C.; Morgan, M. Implant retained overdentures for two patients with severe lichen planus: A clinical report. *J. Prosthet. Dent.* **2003**, *89*, 6–10.
15. Czerninski, R.; Eliezer, M.; Wilensky, A.; Soskolne, A. Oral lichen planus and dental implants—A retrospective study. *Clin. Implant. Dent. Relat. Res.* **2013**, *15*, 234–242.
16. Moergel, M.; Karbach, J.; Kunkel, M.; Wagner, W. Oral squamous cell carcinoma in the vicinity of dental implants. *Clin. Oral Investig.* **2014**, *18*, 277–284.
17. Aboushelib, M.N.; Elsafi, M.H. Clinical Management Protocol for Dental Implants Inserted in Patients with Active Lichen Planus. *J. Prosthodont.* **2017**, *26*, 29–33.
18. Anitua, E.; Piñas, L.; Escuer-Artero, V.; Fernández, R.S.; Alkhraisat, M.H. Short dental implants in patients with oral lichen planus: A long-term follow-up. *Br. J. Oral Maxillofac. Surg.* **2018**, *56*, 216–220.
19. Khamis, A.K.; Aboushelib, M.N.; Helal, M.H. Clinical Management Protocol for Dental Implants Inserted in Patients with Active Lichen Planus. Part II 4-Year Follow-Up. *J. Prosthodont.* **2019**, *28*, 519–525.
20. Mozzati, M.; Gallesio, G.; Menicucci, G.; Manzella, C.; Tumedei, M.; Del Fabbro, M. Dental Implants with a Calcium Ions-Modified Surface and Platelet Concentrates for the Rehabilitation of Medically Compromised Patients: A Retrospective Study with 5-Year Follow-Up. *Materials* **2021**, *14*, 2718.
21. Hernández, G.; Lopez-Pintor, R.M.; Arriba, L.; Torres, J.; de Vicente, J.C. Implant treatment in patients with oral lichen planus: A prospective-controlled study. *Clin. Oral Implant. Res.* **2012**, *23*, 726–732.
22. López-Jornet, P.; Camacho-Alonso, F.; Sánchez-Siles, M. Dental implants in patients with oral lichen planus: A cross-sectional study. *Clin. Implant. Dent. Relat. Res.* **2014**, *16*, 107–115.
